# An Observational Study on the Management of Medico-Legal Maxillofacial Trauma Cases by General Practitioners

**DOI:** 10.3390/healthcare12181803

**Published:** 2024-09-10

**Authors:** Constantin Răzvan Giuvara, Victor Vlad Costan, Otilia Boisteanu, Adina Armencia, Mihai Ciofu, Eduard Radu Cernei, Carina Balcos, Bulgaru Iliescu, Gabriela Calin, Loredana Liliana Hurjui

**Affiliations:** 1 Department of Medical Disciplines, Faculty of Dentistry, “Grigore T. Popa” University of Medicine and Pharmacy, 700115 Iasi, Romania; razvan.giuvara@umfiasi.ro (C.R.G.); victor.costan@umfiasi.ro (V.V.C.); otilia.boisteanu@yahoo.com (O.B.); oanaarmencia@yahoo.com (A.A.); mihai.ciofu@umfiasi.ro (M.C.); cerneiradu@yahoo.com (E.R.C.); loredana.hurjui@umfiasi.ro (L.L.H.); 2 Department of Medical Disciplines, Faculty of Medicine and Pharmacy, “Grigore T. Popa” University of Medicine and Pharmacy, 700115 Iasi, Romania; bulgarudiana@yahoo.com; 3 Faculty of Dentistry, Apollonia University, 700511 Iasi, Romania

**Keywords:** maxillofacial trauma, forensic issues, forensic knowledge, attitudes

## Abstract

Maxillofacial trauma, as seen from a medico-legal point of view, is an integral part of medical practice in emergency departments. Therefore, general practitioners should have sufficient knowledge about their roles and responsibilities in managing these cases. This study aimed to assess general practitioners’ knowledge, practices, and attitudes regarding managing medico-legal cases (MLCs). Material and method: This study included 113 general practitioners from St. Spiridon Hospital in Iasi, Romania. Participants completed a self-administered structured questionnaire assessing the knowledge, attitudes, and practices of general practitioners regarding the handling of medico-legal cases. Results: The scores obtained for the level of knowledge, attitudes, and practices indicate a good level of knowledge on the part of the responding doctors, with the average value being 38, in a range from 0 to 49 (min. value 28–max wave. 47). The score regarding the attitudes of doctors related to the management of medico-legal cases is modest, with the average value being 37 points out of a maximum of 60 points (min. 14–max. 51). The same situation is recorded in the case of practices regarding the management of medico-legal cases, with the average value being 68 out of a maximum value of 90 (min. 38–max. 84). Conclusion: This study’s results revealed the absence of a well-defined protocol for the recognition and handling of medico-legal cases among general practitioners from Iasi and the need to improve the level of attitudes and practices regarding the management of medico-legal cases. The limitations of this study included the relatively small sample from a single hospital and the use of a methodology based on self-administered questionnaires, which may be subjective. Accordingly, future studies should involve larger and more diverse samples to monitor changes in knowledge and practices over time and qualitative methodologies to gain deeper physician-related insights into medical case management.

## 1. Introduction

Documentation is an essential part of healthcare delivery. It helps healthcare providers communicate, plan, and evaluate patient treatment, create permanent records for future care, and establish databases to assess treatment effectiveness and facilitate research. A well-documented chart will confirm the continuity of care. In medico-legal cases, accurate and complete medical records are essential as they provide vital evidence to establish facts, determine liability, and evaluate the standard of care provided. These records offer detailed information about the patient’s condition, treatments, and any complications or side effects [1].

The knowledge and attitudes of healthcare personnel in managing medico-legal records are crucial for maintaining accurate and reliable documentation in healthcare settings. A solid understanding of medical records’ legal implications and requirements is necessary. This involves familiarity with policies, regulations, and best practices related to record keeping, confidentiality, and data protection. With the necessary knowledge, patient information, treatment plans, and any significant events or incidents can be accurately recorded. In addition to knowledge, a proactive, responsible attitude toward forensic cases is necessary [2]. They should recognize the importance of these records in ensuring patient safety, facilitating effective communication among healthcare professionals, and providing crucial evidence in legal proceedings.

A medico-legal case (MLC) refers to an injury or condition that involves collaboration between medical personnel and qualified law enforcement personnel to establish criminal liability for the cause under the law of the respective country [3].

MLCs are an integral part of medical practice in terms of causality. This points to the important role of the physician as the first person to assess injuries before they are altered by surgery or healing. General practitioners therefore have a responsibility to provide relevant medical information to the judiciary, which will be legally relevant [4]. In addition, forensic cases can be substantiated in primary medical reports without involving forensic experts. This reveals the seriousness and importance of documentation by the doctor, as this may be the only valid document before the legislator.

Maxillofacial trauma has particular forensic implications and is considered a forensic case that meets the criteria for polytrauma. Maxillofacial injuries are frequently encountered in emergency medicine; most patients with these injuries have multiple system traumas requiring coordinated management between emergency general practitioners and specialists in maxillofacial surgery, otolaryngology, plastic surgery, and general surgery [5,6,7,8,9].

This type of trauma requires special attention because there are multiple important sensory systems (olfactory, auditory, somatic, gustatory, and vestibular) at this level. Vital structures located in the head and neck region (airways, blood vessels, and nerves) are associated. Ultimately, the psychological impact of these injuries can be devastating [8,9,10]. A patient with maxillofacial trauma is a polytrauma patient who requires careful and regularly updated assessments involving interdisciplinary collaboration.

This is because a preoperative functional area reconstruction plan is necessary to guide the physical, psychological, and social rehabilitation process [8,9]. For these reasons, this study aimed to assess the knowledge, practices, and attitudes of general practitioners regarding the handling of medico-legal cases.

## 2. Materials and Methods

This observational study was conducted among a group of 113 general practitioners from St. Spiridon Hospital in Iasi, Romania, who, after being informed about the content of the study, signed an informed consent and answered a self-administered structured questionnaire assessing the knowledge, attitudes, and practices of general practitioners regarding the handling of medico-legal cases.

The questionnaire included four sections (the first section grouped the characteristics of the responding general practitioners (age, gender, job title, specialty, education, and length of work experience).

The second section included 49 questions assessing doctors’ knowledge of the correct handling of forensic cases (definition and examples of forensic cases, ethical and legal issues, writing a forensic medical report (MLR), and handling forensic or legal evidence). Correct answers were marked with 1 mark and incorrect answers were marked with 0 marks “not correct” answers were included in the incorrect category. The score that can be obtained in this section ranges from 0 to 49.

The third section contained items measuring general practitioners’ attitudes toward treating MLCs (12 items). Participants were asked to respond on a Likert scale from 0 to 5 whether they “strongly agree”, “agree”, “have no opinion”, “disagree” or “strongly disagree” with the statements. For each item, 5 points were given for “strongly agree” to 1 point for “strongly disagree”, and vice versa for negative statements. Thus, high scores reflected positive attitudes and low scores reflected negative attitudes (the potential range was 12–60).

The fourth section consisted of 30 points for assessing doctors’ practice in dealing with forensic cases. This section includes knowledge of notifying authorities about MLCs, keeping forensic evidence, and documenting and writing MLRs. The response to each item was coded on a Likert scale from 0 to 3, with 3 points awarded for “Always”, 2 points for “Sometimes” and 1 point for “Never” Negative statements were scored inversely. Thus, a score of 30 indicated the most undesirable practice, while 90 indicated the best practice in handling MLCs (3).

The collected data were coded and processed with the help of IBM SPSS statistics software version 26.0 (IBM, Armonk, NY, USA). The t-test was used to test for significant differences in the measured scores and *p* < 0.01 was statistically significant.

## 3. Results

Of the 113 doctors who participated in this study, 47.8% are over 40 years old, and 18.6% are between 31 and 40 years old. Moreover, 65.5% of the participants are male subjects. In addition, 76% of the respondents are already specialists, 73.5% have postgraduate studies and 45.1% have more than 10 years of professional experience (Table 1). A significant statistical difference can be observed between the resident practitioners and the specialists, *p* < 0.01.

**Figure 1:**
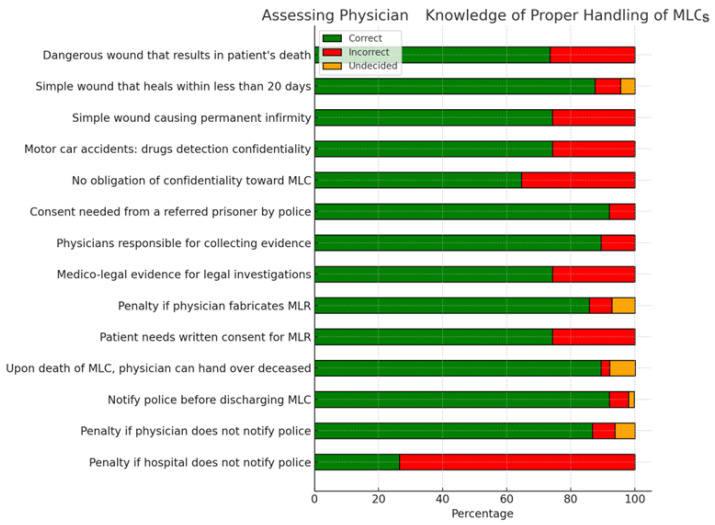
Distribution of answers related to assessing physicians’ knowledge of proper handling at the MLC level.

Figure 1 shows the distribution of responses to the statements related to the level of knowledge regarding the proper handling of MLCs. The results show us that the doctors know how to evaluate medico-legal cases, they see the collection of medico-legal evidence, collaborate with the police when necessary, as well as the types of injuries depending on their severity, and injuries that have medico-legal implications.

**Figure 2:**
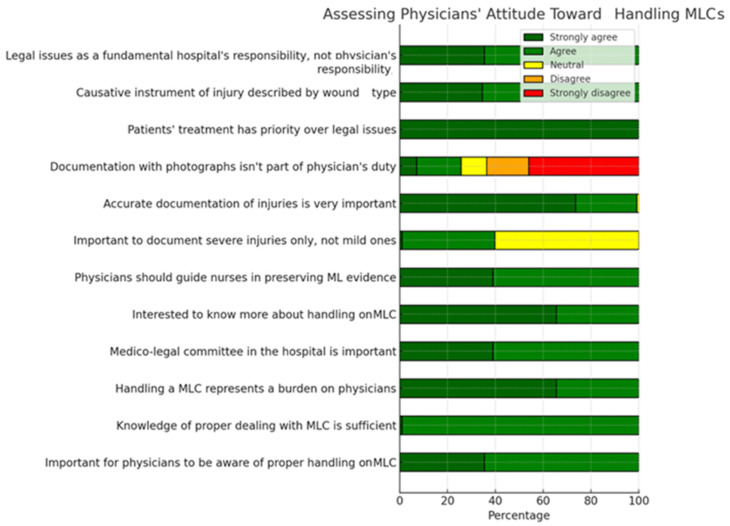
Distribution of answers to the statements assessing general practitioners’ attitudes toward handling MLCs.

The assessing general practitioners’ attitudes toward handling MLCs shows us that although they consider that they have sufficient knowledge regarding the management of medico-legal cases, doctors declare that the preparation of medico-legal documentation is an additional, unpleasant, task for which the hospital institution should be responsible, not the doctor. At the same time, doctors are aware that all injuries, regardless of the severity, must be described in detail and that the samples must be collected in their entirety but not kept by the doctor, who must give priority to the treatment and not to the medico-legal aspects (Figure 2).

**Figure 3:**
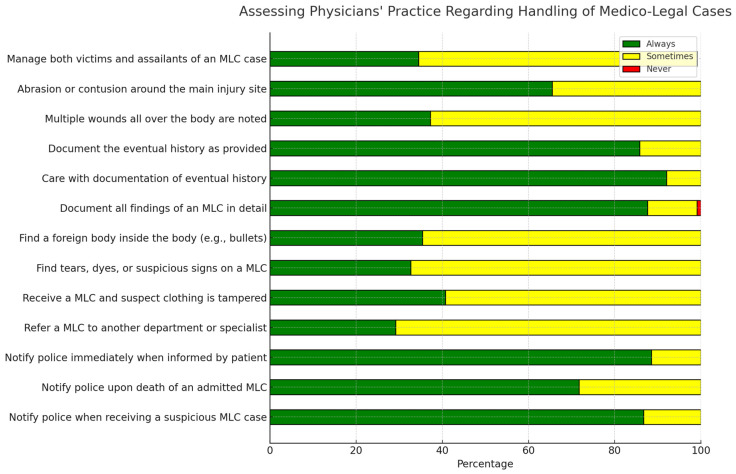
Distribution of answers to the statements assessing general practitioners’ practices regarding the handling of an MLCs.

The distribution of answers to the statements assessing general practitioners’ practices regarding the handling of MLCs shows participants’ responses to items assessing their practices regarding the handling of MLCs. The results presented in Figure 3 show us that the doctors who draw up MLRs insist a lot on the history of the accident and the gathering of all the evidence, but less on some details, such as the number of injuries on the body (37.2%), the description of the contusions (65.5%), keeping the body clean inside the body (e.g., bullet, shots, broken blade of weapon) (35.4%), and keeping textile articles (32.7%). Instead, there is a tendency to report possible medico-legal situations to the police (suicides, suspicious deaths, armed attacks).

The scores obtained for the level of knowledge, attitudes, and practices are described in Table 2. The knowledge score indicates a good level of knowledge among the responding doctors, with the average value being 38, in a range from 0 to 49 (min. value 28–max wave. 47). The score regarding the attitudes of the doctors related to the management of medico-legal cases is modest, with the average value being 37 points out of a maximum of 60 points (min. 14–max. 51). The same situation is recorded in the case of the practices regarding the management of medico-legal cases, with the average value being 68 out of a maximum value of 90 (min. 38–max. 84).

## 4. Discussion

Doctors working in emergency departments are frequently confronted with medicolegal cases (MLCs). Therefore doctors should have sufficient knowledge about their roles and responsibilities regarding this type of trauma to assist justice [11,12,13,14]. This study found that participants did not have the necessary knowledge, so they resorted to guidance on handling MLCs. Similar findings were reported by Wong, who surveyed doctors working in Hong Kong; he showed that the majority of respondents had no training in forensic medicine [15]. The significance of ongoing education in forensic medicine, focusing on data from a study revealing significant knowledge and practice deficiencies among participating doctors in managing medico-legal cases, underscores the critical necessity of enhancing education and training in this area.

In terms of the respondents’ attitudes toward MLCs, most of the respondents agreed with the importance of physician awareness of the proper handling of MLCs; approximately 99% of participants considered the level of knowledge on this issue to be sufficient. These findings were consistent with similar studies in the literature, showing that many practitioners were not very confident about forensic issues [15,16,17,18].

Moreover, 64.4% of participants considered legal issues to be a fundamental responsibility of the hospital and not the doctor. A study conducted in the Netherlands reported similar results, showing that 75% of practitioners consider the assessment and description of abuse-related injuries to be outside the responsibility of the treating physician [3].

From a forensic point of view, it is very important to examine and document injuries around the main one, as it can help either to identify or to differentiate the causative agents. Therefore, all injuries, even those that appear insignificant, should be recorded [5].

If the protocol for treating MLCs was part of the university curriculum, the level of knowledge of doctors would be significantly higher than that of other doctors. However, the attitudes and practices of the study participants do not differ significantly from other results reported in the literature; they can be explained by the effect of a working environment in which knowledge, attitudes, and practices can be acquired from the hospital medical staff.

Residents performed worse than interns in their understanding of forensic case management (mean score of 28 versus 40). This important differentiation suggests that increased clinical experience and continued education contribute to increased preparedness for these circumstances. Physicians with postgraduate training demonstrated a more positive response toward the management of medico-legal cases (average score of 45) than the university-educated doctors did (average score of 30). Through this research, additional education can improve attitudes as well as knowledge regarding the importance of precise documentation and cooperation with law enforcement.

According to the data, doctors with less than five years of experience and those with more than ten years differ significantly in their practices. The more demanding and specialized training programs early in their careers are highlighted by the fact that those in the first category expressed greater insecurity when handling forensic evidence and when properly documenting cases.

To serve the legal system, doctors working in emergency departments must be properly educated about their roles and obligations when treating medico-legal cases (MLCs) [19,20,21,22,23,24]. This study shows that the participants lacked the requisite understanding and frequently required assistance in handling MLCs. Wong revealed similar results in a poll of Hong Kong doctors, revealing that the majority of participants lacked formal training in legal medicine [15].

Medical professionals in the oral and maxillofacial surgery sector dealing with facial injury treatment should be responsible for repairing the aesthetic defect (surgical or conservative), restoring functions, and reducing the disability period. However, a study conducted by Vrinceanu in 2014 reported a higher percentage of surgically treated cases [16,25]. According to Perry and Hailemichael, approaches for craniomaxillofacial fractures follow clinical guidelines, with non-displaced fractures managed conservatively for functional and aesthetic outcomes, and displaced fractures managed surgically through open reduction and internal rigid fixation with mini plates [12,24].

Some studies have tried to find the relationship between head injuries and maxillofacial injuries. Therefore, emergency doctors should make their clinical decisions only after a careful assessment of patients. The medical-legal requirements of patients should be addressed correctly by the medical team [23,26,27,28]. The avoidance and fear of dealing with MLCs by doctors are well documented. Therefore, regardless of their specialty, obtaining adequate knowledge in the field of legal medicine is crucial for all medical practitioners to overcome their fear of MLCs [11,29,30].

On the other hand, primary medical reports can represent the sole technical document on which the court relies, without the need for verbal testimony from medical professionals. This underscores the importance of meticulous documentation. There is a significant discrepancy between hospitals and forensic medical reports worldwide, possibly due to the medical team’s focus on saving patients’ lives, leading to limited documentation in tense situations [17,19].

The concept of trace evidence, developed by Edmund Locard in 1920, has transformed how law enforcement and scientists handle criminal investigations. According to Locard, the absence of evidence at a crime scene should be unimaginable. Medical professionals can search for forensic evidence by conducting a physical examination and collecting biological samples from the patient’s body. Health professionals have a relatively high probability of encountering medical-legal cases, but such situations are often ignored. Saving the patient’s life is the doctors’ primary objective. Most doctors are cautious when handling medical-legal cases, either trying to avoid or quickly resolve them due to fear. Every doctor should be aware that their experience and services may be required by the justice department [31,32,33]. Because legal systems differ by country and accurate documentation of a patient’s initial examination is vital in starting the legal process, it is essential for doctors to be proficient in clinical skills and specific legal procedures. Our research underscores the necessity for more educational programs and ongoing training in forensic medicine.

Research conducted by Mostafa and his team revealed that Egyptian doctors lack sufficient knowledge and proper skills in terms of handling medico-legal cases, underscoring the importance of ongoing training programs in forensic medicine [2]. Collectively, 50.3% of participating doctors had reasonable knowledge, while only 16.6% had good knowledge. Regarding attitude, 67.6% had a positive attitude, but only 5.5% had good practice. A lack of formal education and training courses in legal medicine at the postgraduate level was highlighted as a common issue among most doctors [22].

Similar results have been reported in studies conducted in Egypt, Saudi Arabia, and Hong Kong [34]. Pre-university medical schools include legal medicine in their curriculum but without practical field training. After graduation, doctors do not receive mandatory training in legal medicine in their clinical specialties [16,20].

Although doctors understand the importance of photographic documentation, only a small percentage of this study carried out photographic documentation. Obtaining patient consent for photography documentation is crucial for legal purposes. Therefore, measures should be taken in medical facilities to ensure adequate photographic documentation. Training in forensic photography should be included in a clinical legal medicine training course for emergency department residents, and doctors should receive training in forensic photography.

Dealing with patients’ relatives is another problem frequently encountered by participants. This may be due to the absence of a well-defined policy establishing or delegating legal responsibilities to doctors. In addition, they may feel obliged to confront patients’ relatives without adequate security measures. The lack of these is a well-known problem and sometimes leads to members of the medical team being exposed to violence. The risk of exposure to violence is exaggerated in forensic cases as they include domestic violence cases and assault victims.

An insufficiently reasoned medico-legal report is an important problem due to insufficient documentation of all the details of patient management [3].

Incorporating clinical forensic medicine into regular forensic procedures can greatly improve the quality and precision of medico-legal evaluations. This methodology guarantees the proper documentation and interpretation of clinical results within the framework of legal norms, resulting in more dependable conclusions in forensic situations [33]. For physicians to properly participate in legal investigations, they must have forensic medicine education and training. Healthcare professionals can more effectively interact with forensic experts and comprehend the legal ramifications of their results by bridging the gap between clinical practice and forensic pathology [34]. It has been demonstrated that integrating forensic medicine into clinical settings enhances patient outcomes by guaranteeing that injuries are carefully assessed for possible legal ramifications. This strategy ensures that all the pertinent elements are taken into account, which benefits the legal system as well as the healthcare system [34].

## 5. Conclusions

Dental jurisprudence is a critical and heavy subject. Injuries are complex and interpreting them correctly demands the utmost objectivity, observation skills of an unprecedented caliber, analytical powers that have been seldom seen previously in this sport, and above all, integrity. The knowledge is reasonable but the attitudes and practices related to managing medico-legal cases are poor, which should be improved with educational programs. To contribute to filling these gaps, future studies would do well to investigate certain specific themes, like the influence of ongoing education on legal medicine, interdisciplinary work regarding maxillofacial trauma management, and psychological dimensions of general practitioners’ practice with medico-legal cases.

## Figures and Tables

**Table 1:** Demographic characteristics of the study group.

**Age**	**Percent**	**N**	**Mean**	**SD**	***p*-Value**
31–40 years	18.6%	21	36.609	4.568	0.00
>40 years	47.8%	54	56.701	6.452	
**Gender**		**N**	**Mean**	**SD**	***p*-Value**
Female	34.5%	39	8.125	7.1839	0.9134
Male	65.5%	74	67.95	5.3645	
**Type of General Practitioners**		**N**	**Mean**	**SD**	***p*-Value**
Resident	23.9%	27	38.92	2.976	0.0043
Specialist	76.1%	86	54.43	2.785	
**Studies**		**N**	**Mean**	**SD**	***p*-Value**
University studies	26.5%	30	37.77	3.145	0.0001
Post-university studies	73.5%	83	57.32	2.987	
**Years of Practice**		**N**	**Mean**	**SD**	***p*-Value**
5–10 years	28.3%	32	31.55	3.112	0.0134
>10 years	45.1%	51	63.87	4.563	

**Table 2:** The scores obtained for the level of knowledge, attitudes, and practices regarding the management of medico-legal cases.

	No of Items	Score	Results
Knowledge Score	14	0–49	38
			(min. 28–max. 47)
Attitude Score	12	12–60	37
			(min. 14–max. 51)
Practice Score	30	30–90	68
			(min. 38–max. 84)

## Data Availability

The original contributions presented in the study are included in the article, further inquiries can be directed to the corresponding authors.
